# To call or not to call: exploring the validity of telephone interviews to derive maternal self-reports of experiences with facility childbirth care in northern Nigeria

**DOI:** 10.1136/bmjgh-2021-008017

**Published:** 2022-03-16

**Authors:** Nasir Umar, Joanna Schellenberg, Zelee Hill, Antoinette Alas Bhattacharya, Moise Muzigaba, Özge Tunçalp, Nuraddeen Umar Sambo, Abdulrahman Shuaibu, Tanya Marchant

**Affiliations:** 1 Department of Disease Control, Faculty of Infectious and Tropical Diseases, London School of Hygiene & Tropical Medicine, London, UK; 2 Institute for Global Health, Department of Epidemiology and Public Health, University College London Research, London, UK; 3 Department of Maternal, Newborn, Child and Adolescent Health and Ageing, World Health Organization, Geneve, Switzerland; 4 Sexual and Reproductive Health and Research, World Health Organizations, Geneva, Switzerland; 5 Data Research and Mapping Consult Limited, Abuja, FCT, Nigeria; 6 Executive Office, State Primary Health Care Development Agency, Gombe, Nigeria

**Keywords:** maternal health, health services research, public health, epidemiology

## Abstract

**Background:**

To institutionalise respectful maternity care, frequent data on the experience of childbirth care is needed by health facility staff and managers. Telephone interviews have been proposed as a low-cost alternative to derive timely and actionable maternal self-reports of experience of care. However, evidence on the validity of telephone interviews for this purpose is limited.

**Methods:**

Eight indicators of positive maternity care experience and 18 indicators of negative maternity care experience were investigated. We compared the responses from exit interviews with women about their childbirth care experience (reference standard) to follow-up telephone interviews with the same women 14 months after childbirth. We calculated individual-level validity metrics including, agreement, sensitivity, specificity, area under the receiver operating characteristic curve (AUC). We compared the characteristics of women included in the telephone follow-up interviews to those from the exit interviews.

**Results:**

Demographic characteristics were similar between the original exit interview group (n=388) and those subsequently reached for telephone interview (n=294). Seven of the eight positive maternity care experience indicators had reported prevalence higher than 50% at both exit and telephone interviews. For these indicators, agreement between the exit and the telephone interviews ranged between 50% and 92%; seven positive indicators met the criteria for validation analysis, but all had an AUC below 0.6. Reported prevalence for 15 of the 18 negative maternity care experience indicators was lower than 5% at exit and telephone interviews. For these 15 indicators, agreement between exit and telephone interview was high at over 80%. Just three negative indicators met the criteria for validation analysis, and all had an AUC below 0.6.

**Conclusions:**

The telephone interviews conducted 14 months after childbirth did not yield results that were consistent with exit interviews conducted at the time of facility discharge. Women’s reports of experience of childbirth care may be influenced by the location of reporting or changes in the recall of experiences of care over time.

**Key questions**

**What is already known?**

- Frequent data are needed to promote positive childbirth experience in health facilities, but conventional data collection methods, including face-to-face household surveys or exit interviews, are resource intensive limiting their routine use in low-income and middle-income countries.

**What are the new findings?**

- We explored the validity of telephone interviews to derive estimates of respectful maternity care and observed similar demographic characteristics between the women interviewed at the time of facility discharge and those reached by telephone interview.
- However, women’s answers given at exit interview and subsequent telephone interview 14 months after childbirth about positive and negative maternity care experiences diverged.

**What do the new findings imply?**

- Our findings suggest that telephone interviews can be used to reach women who access facility-based childbirth care.
- Measures of women’s experience of facility-based childbirth care may change over time, by location, or by data collection method and more studies are needed to explore this further.

## Introduction

This study complements the ongoing global response towards eliminating mistreatment during facility-based childbirth and the institutionalisation of respectful maternity care.^1 2^ The global commitment to improve positive experience during labour and childbirth has resulted in a renewed impetus to typify positive and negative experiences during facility-based childbirth, and develop tools to capture and evaluate women’s experiences following facility-based maternity care.^3–11^

In low-income and middle-income countries (LMIC) such as Nigeria, quantitative data on the reported experience of care are mostly sourced through face-to-face community surveys or exit surveys.^12–14^ These methods are resource intensive and are typically applied in research settings, mostly as standalone and cross-sectional studies, and do not apply harmonised measurement tools.^12 15^ To have valid, timely and actionable data on the experience of childbirth care, novel methodologies need to be explored.

The proliferation of mobile phone ownership in LMIC presents an opportunity to transform the current methods of data collection via telephone interviews.^15 16^ Mobile phones are the fastest adopted technologies in recent times, and ownership cuts across all socioeconomic levels.^15 17^ Mobile phone spread per unique user, a good proxy for mobile phone ownership, is moderate to high in LMIC: 87% in Kenya, 84% in South Africa, 74% in Ghana and 64% in Nigeria.^17^

Compared with face-to-face survey methods, using telephone interviews to collect data from communities could considerably increase the timeliness and reach of data while also reducing cost and logistical challenges.^15 16 18^ But they may also introduce selection bias (if phone interviews under-represent people of lower education or economic status) and social desirability bias (if respondents prefer not to answer sensitive questions over the phone).^19 20^ Other possible issues might be reduced credibility of telephone interviewers and respondents being more easily distracted when answering questions over the phone.

There is currently a dearth of evidence on the validity of telephone interviews to derive estimates of respectful maternity care. This study reports the validity of experience of care responses derived from telephone interviews with women who had a childbirth in health facilities in northern Nigeria, when compared with their responses during facility exit interviews.

## Methods

### Study setting

Gombe State, the study setting, is one of the 36 states of the Federal Republic of Nigeria, located in the country’s North-East region. Gombe State has an estimated population of 2.6 million, based on population projections from the 2006 national census. About 75% of the state is rural, with a high fertility rate of 7.0 live births per 1000 females aged 15–49. Service utilisation for maternal and newborn health services is low: for example, only 44% of pregnant women sought 4 or more antenatal care visits in 2019, only 28% had a facility-based childbirth and only 21% of the deliveries were conducted by a skilled birth assistant.^21–23^

### Indicator selection

We collected data on 26 experience of maternity care indicators focusing on 8 positive maternity care experiences and 18 negative maternity care experiences. The negative maternity care experience indicators were drawn from the typology of mistreatment, which included domains of physical abuse, verbal abuse, sexual abuse, stigma and discrimination, failure to meet professional standards of care, poor rapport and communication between women and providers, and health systems conditions and constraints.^12^ We referred to the literature on improving quality of maternal and newborn care in health facilities and the earlier literature assessing experience of childbirth care to identify the eight positive maternity care experience indicators (ie, practices that recognise women’s preferences and needs).^7 13 24–26^ The research team agreed on the final list of indicators described in online supplemental table S1 through discussion and consensus.

10.1136/bmjgh-2021-008017.supp1 Supplementary data

### Data collection

The study was nested within a programme of work aimed at understanding the quality of maternal and newborn care in Gombe State, Nigeria.^27^ We collected exit interview data from mothers in 10 primary healthcare (PHC) facilities, in Gombe State, in August–September 2019. Mothers were eligible and invited for the exit interviews if they were discharged (usually within 24 hours of childbirth) with a live baby following facility-based childbirth and provided informed consent to participate in the study. The exit interviews were conducted in Hausa. The exit interview instrument covered demographic information of study participants, the content of care provided to the mother and the newborn, and experiences of facility-based childbirth care. Women were also asked about their access to mobile phones and, for those with access, permission to make a follow-up call in the future was solicited.

In October–November 2020, we conducted telephone interviews with the same mothers surveyed during exit survey. Only mothers that participated in the exit interviews, provided telephone numbers and consent were included in the follow-up telephone interviews. In both exit interviews and telephone interviews, mothers were asked the same questions about their experience of facility-based childbirth care (online supplemental table S1), with responses to questions dichotomised as ‘experienced an event’ (yes) and ‘not experienced an event’ (no).^28^

All interviewers for both exit and telephone interviews were from Gombe State and were trained in-house for 5 days to familiarise themselves with the questionnaires and data collection procedures, followed by a full pilot and refinement of the study tool. To ensure confidentiality, all the exit interviews were conducted in an area reserved for the interviews or in a separate room within the health facilities. For the telephone interviews, women were encouraged to find a quiet place at home conducive for the telephone interview. The exit interview data were collected in 10 facilities, with 2 trained data collectors and a supervisor working in shifts covering day and night deliveries, 7 days a week for approximately 4 weeks. The telephone interviews were completed in 2 weeks by three data collectors conducting approximately 10 telephone interviews per day. In both the exit and telephone interviews, women were assured that any information collected about them would be kept private and that all data including name, phone number their contact details and interview answers would be fully anonymised.

### Sample size

A minimum sample size of 294 women interviewed at exit and at follow-up telephone interviews was estimated to be adequate to estimate sensitivity, specificity and AUC as an overall index of accuracy. This estimate was based on 50% prevalence of indicators from exit interviews (reference standard) and a set sensitivity of 75%±7% precision, specificity of 75%±7% precision, type 1 error of 0.05, assuming a normal approximation to a binomial distribution.^29^

### Statistical analysis

Exit survey and the telephone interviews were matched by unique participant id. All analyses were conducted using STATA V.16 (www.stata.com). For the validation analysis, exit survey measures of positive and negative maternity care experiences were used as the reference standard and compared with telephone interview responses with the same mothers.

We tabulated the mother’s characteristics at exit survey (all women interviewed without a mobile phone) and follow-up telephone interview to compare demographics and childbirth environment characteristics. We determined the prevalence of positive and negative maternity care experiences for each indicator by the measurement method. Exit interview and telephone interview responses were cross tabulated to construct two-by-two tables, excluding any do not know responses. We calculated per cent agreement between the exit and the telephone interviews. We calculated the sensitivity (true positive rate) and specificity (true negative rate) for each indicator. We quantified the area under the receiver operating characteristic curve (AUC) and estimated 95% CI assuming a binomial distribution. Because this study population included a large number of women with no formal education, we explored the association of educational status (not educated/ educated) of mothers with their reporting consistency for positive maternity care experience measures using the rocreg command in STATA.^30^ Consistent with the recommendation by Munos *et al,*^31^ indicators with very low or very high prevalence, that resulted in fewer than five counts per cell in the two-by-two tables, were included in tabulations for transparency but cannot be interpreted with confidence. An AUC value of 0.5 reflects a random guess while 1.0 reflects perfect accuracy.^31^ We presented findings below in line with Strengthening the Reporting of Observational Studies in Epidemiology statement.^32^

### Patient and public involvement

A preliminary consultation with a different set of women was conducted prior to the main telephone interviews to pretest the telephone interview protocol for appropriateness and understanding. We asked the respondents for feedback about the telephone interview procedures including perceived difficulty, compatibility and clarity of instructions. We used respondent’s inputs to refine the telephone interview protocol.

## Results

Results on the sample characteristics, reported facility childbirth experience in exit and telephone interviews, validation of positive and negative maternity care experience measures are presented below.

### Sample characteristics

**Figure 1:**
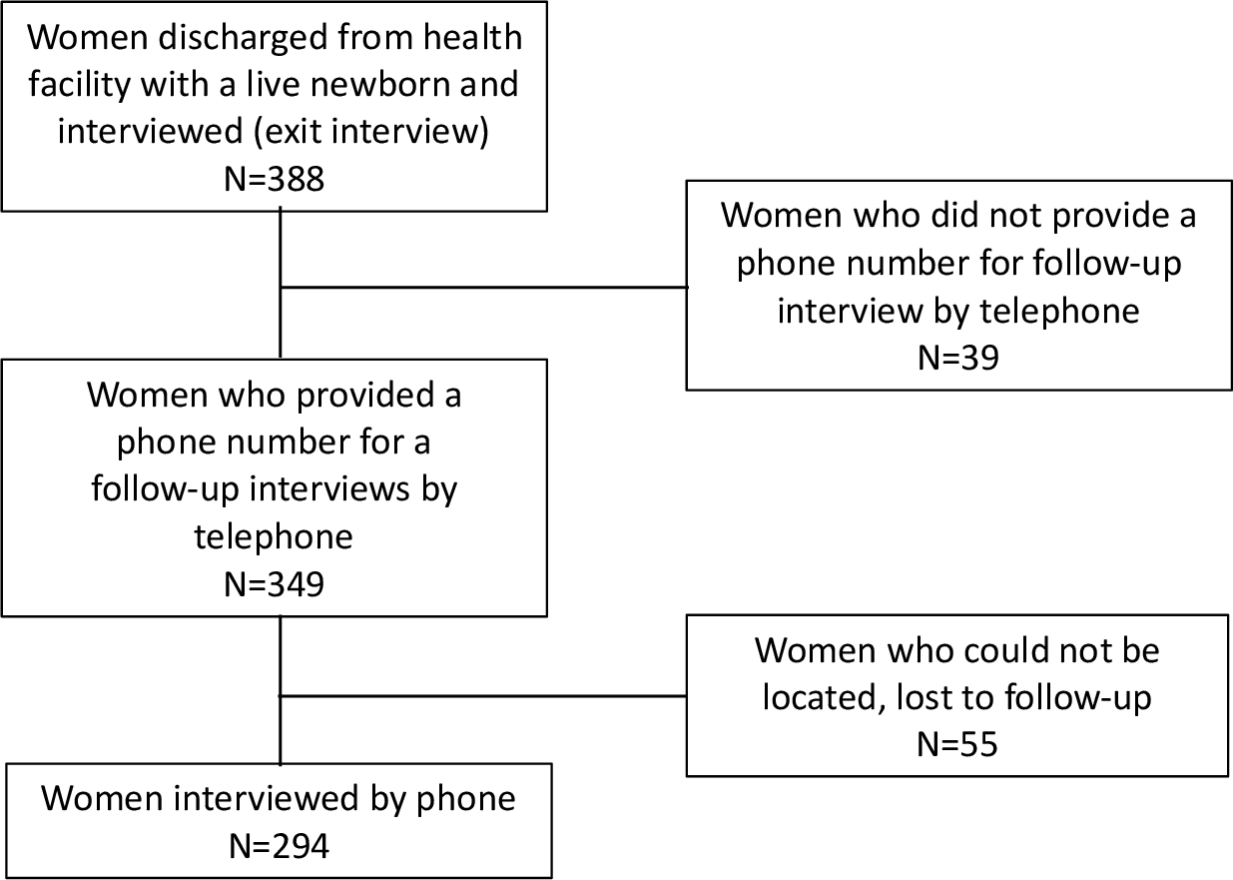
Flow of participants through enrolment at exit interview and follow-up through telephone interview.

A total of 388 mothers participated in the exit survey, 349 of whom provided telephone numbers and agreed to a follow-up telephone interview; subsequently 294 mothers were successfully interviewed 1 year later by telephone and none of the women reached refused to participate (figure 1). From the exit survey, mother’s age at the time of childbirth ranged from 15 to 43 years, with a median of 25 years of age (IQR: 20–30 years). Approximately 50% of the mothers had at least four prior births, about 40% of mothers had no formal education, and 99% were married. About 66% of births were attended by a community health extension worker (CHEW) or junior CHEW (table 1). Demographic characteristics were similar between the original exit interview group (n=388) and those reached in followed-up telephone interview (n=294).

**Table 1:** Characteristics and delivery setting of respondents in exit interviews and telephone follow-up interviews

	Exit interview N=388, N (%)	Telephone interview N=294, N (%)	χ^2^ p value
Age of woman at delivery			
15–19	44 (11)	31 (11)	
20–24	138 (36)	102 (35)	
25–29	108 (28)	85 (29)	
30–34	55 (14)	44 (15)	
>34	43 (11)	32 (10)	0.999
Prior parity			
0	76 (20)	53 (18)	
1–3	120 (31)	92 (31)	
4 or more	192 (49)	149 (51)	0.869
Educational attainment			
None	167 (43)	114 (39)	
Primary	78 (20)	60 (20)	
Secondary	128 (33)	106 (36)	
Higher	15 (4)	14 (5)	0.827
Time of delivery			
Day, 08:00–18:59	238 (61)	174 (59)	
Night, 19:00–07:59	150 (39)	120 (41)	0.682
Day of delivery			
Weekday	289 (74)	221 (75)	
Weekend	99 (26)	73 (25)	0.820
Main provider during labour and delivery			
Doctor, nurse, or midwife	59 (15)	48 (16)	
Community health extension worker (CHEW), junior CHEW	257 (66)	194 (66)	
Hospital assistant	18 (5)	10 (3)	
Other facility staff	37 (10)	26 (9)	
Other non-staff, including traditional birth attendant	17 (4)	16 (5)	0.875

### Positive maternity care experience measures

For the exit interviews, the prevalence of positive maternity care events (ie, practices that recognise women’s preferences and needs) ranged between 55% for the indicator ‘Were the steps involved in every examination during labour and delivery explained to you?’ and 92% for the indicator ‘Were you respectfully greeted by health workers when they first saw you?’ From the telephone interviews, the range in prevalence was 63%–98%. The only exception was ‘Were you asked which position you would like to deliver in?’ which had lower reported levels: 30% in the exit and 29% in the telephone interviews (table 2).

**Table 2:** Validation of women’s self-reports of positive maternity care experiences comparing telephone interview responses to exit interviews as reference standard

Exit interview (N=388)	Telephone interview (N=294)		Matched pairs						
Prevalence (95% CI)	Prevalence (95% CI)	Do not know (%)	N	Agreement (%)	<5 counts per cell	Sensitivity (95% CI)	Specificity (95% CI)	AUC (95% CI)	ROC reg† (β 95% CI)
Were you respectfully greeted by health workers when they first saw you?									
92 (82 to 97)	99 (95 to 100)	0	294	92	Yes	99 (97 to 100)	0 (0 to 16)	0.49 (0.49 to 0.50)	–
Were you encouraged to have some light food during labour and delivery?									
91 (83 to 96)	66 (60 to 72)	0	293	66	No	68 (62 to 73)	48 (28 to 69)	0.58 (0.48 to 0.68)	0.39 (–0.03 to 0.83)
Were you encouraged to move and change position during labour?									
79 (59 to 91)	71 (63 to 78)	0	294	63	No	72 (66 to 78)	30 (19 to 43)	0.51 (0.44 to 0.57)	−0.24 (0.50 to 0.03)
Did you have a support person present during labour and childbirth?									
69 (49 to 84)	89 (84 to 92)	0	293	65	No	88 (83 to 92)	8 (3 to 16)	0.48 (0.44 to 0.52)	−0.12* (–0.25 to 0.002)
Were you encouraged to have a support person present during labour and childbirth?									
68 (45 to 84)	81 (74 to 87)	0	294	60	No	80 (74 to 85)	17 (10 to 26)	0.49 (0.44 to 0.53)	0.06 (–0.11 to 0.23)
Were you encouraged to ask any questions?									
58 (43 to 72)	64 (55 to 72)	1	291	52	No	35 (27 to 44)	64 (56 to 71)	0.49 (0.44 to 0.55)	0.10 (–0.08 to 0.27)
Were the steps involved in every examination during labour and delivery explained to you?									
55 (42 to 68)	63 (56 to 70)	2	288	50	No	63 (55 to 70)	34 (26 to 43)	0.48 (0.43 to 0.54)	−0.05 (−0.23 to 0.12)
Were you asked which position you would like to deliver in?									
30 (12 to 57)	29 (22 to 37)	0	293	60	No	33 (23 to 44)	72 (65 to 78)	0.52 (0.46 to 0.58)	−0.17** (–0.30 to −0.05)

**p<0.01, *p=0.05.

†ROC regression coefficient for relationship with educational status of mother (note educated as constant).

AUC, area under the receiver operating characteristic curve.

Across positive maternity care experience indicators, agreement between the exit and the telephone interviews ranged between 50% and 92% (table 2). Seven indicators had enough data to calculate AUC and among these the sensitivity of the telephone interviews ranged between 33% and 88%, the specificity ranged between 8% and 72%, while the AUC ranged between 0.48 and 0.58, reflecting very poor validity overall. The effect of mother’s education on reporting consistency was a 12% (on whether women had a support person present during labour and childbirth) and a 17% (on whether women were asked which position they would like to deliver in) reduction in agreement between reference standard and repeat interview for educated women relative to non-educated women.

### Negative maternity care experience measures

At exit interview, 15 of the 18 negative maternity care experience indicators had reported prevalence lower than 5% and had fewer than five counts per cell in the two-by-two tables. For all these indicators, the per cent agreement during phone interview was high at over 80%. For 12 of these 15 indicators, reported prevalence was higher at the telephone interview than the exit interview, but prevalence remained low and the 95% CIs overlapped for all except the indicator ‘dismissing concerns of women during labour and childbirth’ which increased from 0.1% (95% CI 0 to 2) to 4% (95% CI 3 to 7); and the indicator ‘being detained in a facility for failure to pay for services’ which increased from 0.3% (95% CI 0 to 2) to 8% (95% CI 4 to 16).

Three negative maternity care experience indicators had at least five counts per cell in the two-by-two tables. The percentage of women reporting that their ‘birth attendant did not respect the woman’s choice of preferred birth position’ saw a large increase from 5% (95% CI 2 to 14) at exit to 31% (95% CI 26 to 35) in the telephone interview, with 68% agreement. Reporting *‘*denial or lack of birth companion during childbirth’ increased from 17% (95% CI 8 to 32) of women at exit to 31% (95% CI 25 to 38) at telephone interview, but with overlapping 95% CIs, and with 63% agreement. And the percentage of women reporting a ‘lack of privacy during childbirth’ did not change by method, being 12% (95% CI 2 to 51) at exit and 11% (95% CI 6 to 20) at phone interview, with 80% agreement. For each of these three indicators, as for all other negative maternity care experience measures, results suggest very poor validity of phone interview responses against exit interview responses, with AUC ranging between 0.48 and 0.60 (table 3).

**Table 3:** Validation of women’s self-reports of negative maternity care experiences comparing telephone interview responses to exit interviews as reference standard

Exit interview (N=388)	Telephone interview (N=294)		Matched pairs					
Prevalence (95% CI)	Prevalence (95% CI)	Do not know (%)	N	Agreement (%)	<5 counts per cell	Sensitivity (95% CI)	Specificity (95% CI)	AUC (95% CI)
Denial or lack of birth companions during labour and delivery?								
17 (8 to 32)	31 (25 to 38)	0	293	63	No	32 (19 to 47)	69 (63 to 75)	0.50 (0.43 to 0.58)
Lack of privacy								
12 (2 to 51)	11 (6 to 20)	0	293	80	No	13 (4 to 28)	89 (85 to 93)	0.51 (0.46 to 0.57)
Skilled attendant absent at time of delivery?								
5 (1 to 18)	3 (2 to 5)	1	291	93	Yes	0 (0 to 27)	97 (94 to 99)	0.48 (0.47 to 0.49)
BA did not respect woman choice of preferred birth positions?								
5 (1.6 to 14)	31 (26 to 35)	2	289	68	No	39 (14 to 68)	69 (63 to 75)	0.54 (0.40 to 0.68)
Painful vaginal exams (not acknowledging woman discomfort, pain, or refusal to provide pain relief)?								
4 (1 to 12)	9 (6 to 12)	2	289	89	Yes	25 (6 to 57)	92 (88 to 95)	0.58 (0.46 to 0.71)
Language and interpretation issues during labour and delivery?								
3 (0 to 2)	3 (1 to 12)	0	294	96	Yes	0.0 (0 to 98)	97 (94 to 98)	0.48 (0.00 to 1.00)
Woman being beaten, pushed, pinched, slapped, or poked to facilitate the delivery?								
2 (1 to 4)	3 (1 to 7)	1	292	96	Yes	17 (0 to 64)	98 (95 to 99)	0.57 (0.41 to 0.73)
BA used harsh or rude language, judgmental, or accusatory comments during labour and delivery?								
2 (1 to 3)	5 (2 to 9)	1	292	94	Yes	0 (0 to 60)	95 (92 to 97)	0.48 (0.46 to 0.49)
Poor staff attitudes during labour and delivery?								
2 (0 to 6)	5 (3 to 11)	0	294	94	Yes	25 (1 to 81)	95 (92 to 97)	0.60 (0.35 to 0.85)
BA performed unconsented surgical operations?								
1 (0 to 2)	4 (2 to 7)	1	291	95	Yes	0 (0 to 71)	96 93 to 98	0.48 (0.47 to 0.49)
Woman being physically restrained, tied, or gagged during labour and delivery?								
1 (0 to 2)	0.3 (0 to 3)	0	294	99	Yes	0 (0 to 84)	100 (98 to 100)	0.50 (0.49 to 0.50)
Sexual abuse, touched inappropriately or rape during labour and delivery?								
1 (0 to 2)	1 (1 to 2)	1	291	98	Yes	0 (0 to 84)	99 (97 to 100)	0.50 (0.49 to 0.50)
Woman being discriminated against based on ethnicity/race/religion/income/HIV/age during labour and delivery?								
1 (0 to 2)	0.3 (0 to 3)	0	293	99	Yes	0 (0 to 84)	100 (98 to 100)	0.50 (0.4949 to 0.5016)
Lack of supportive care from health workers during labour and delivery?								
1 (0 to 2)	5 (3 to 8)	0	293	95	Yes	0 (0 to 84)	95 (92 to 97)	0.48 (0.46 to 0.49)
Denial of food, fluids, or mobility during labour and delivery?								
0.3 (0 to 3)	2 (1 to 6)	3	286	97	Yes	0 (0 to 98)	98 (95 to 99)	0.50 (0.00 to 1.00)
Woman detained in a facility for failure to pay for services?								
0.3 (0 to 2)	8 (4 to 16)	1	291	91	Yes	0 (0 to 98)	92 (88 to 96)	0.46 (0.00 to 1.00)
BA used threats of withholding treatment or poor outcomes, blamed woman for poor outcomes to facilitate labour and delivery?								
0.2 (0 to 2)	5 (2 to 9)	1	292	95	Yes	0 (0 to 98)	95 (92 to 97)	0.48 (0.00 to 1.00)
BA dismissed the concerns of woman during labour and delivery?								
0.1 (0 to 2)	4 (3 to 7)	0	293	95	Yes	0 (0 to 84)	96 (93 to 98)	0.48 (0.47 to 0.49)

## Discussion

In this study, we defined data from exit interviews of mother’s self-report of childbirth experience following facility birth in August–September 2019 as the ‘reference standard’ and compared this with data from follow-up telephone interviews of the same mothers a year later and estimated individual-level validity metrics.

We found the demographic characteristics of participants reached by telephone to be comparable to those of women interviewed through the exit survey. This is in line with recent studies showing that mobile phone coverage and ownership have dramatically changed globally in the last decades, allowing for telephone survey methods to generate samples comparable to face-to-face surveys, even in rural settings.^28 33^

Of the eight positive maternity care experience indicators, at exit from facility the majority of women reported receiving each positive practice with the exception of being asked about a preferred delivery position. We discerned no systematic pattern of change in prevalence between the two survey methods. However, for all positive indicators, the telephone interviews did not yield results consistent with exit interviews conducted at the time of facility discharge.

Of the 18 negative maternity care experience indicators, the reported prevalence was generally very low at exit except for 17% of women saying that they were denied or lacked a birth companion during labour and childbirth. Notably, however, four negative maternity care experience indicators increased in prevalence during the telephone survey, including more women feeling that the birth attendant had dismissed their concerns, that they lacked supportive care, that their choice of birth position was not respected, and even that they were retained in the facility for failing to pay for services. The very low reported negative maternity care experience prevalence at exit interview restricted our ability to do a detailed analysis of the validity of responses about negative maternity care experiences, but again we note that the AUC for all indicators was equivalent to that of a random guess.

In interpreting these results, we cannot discount alternative explanations beyond the poor validity of the data collection method itself, including the passing of time, location and whether and how reference standard measures of reported experience can be determined. For example, individual perspective and recall might change over time to become more, or less pronounced as the experienced is processed. We had originally planned for a shorter interval between exit and telephone interviews but were delayed by the emergence of COVID-19: we cannot know what difference this may have made to the results. Place of reporting might also have some influence over measures—women might not be able to report negative maternity care experiences while still in the facilities and feel better able to report negative maternity care experiences when in the safety of their own homes.

Studies on the validity of telephone interviews to derived positive and negative maternity care experience measures are limited and we are not aware of alternative findings from which to draw comparisons. Studies on validity of self-reported facility-based childbirth care conducted face-to-face found mixed results when assessing mothers recall of events that occurred during labour, childbirth and postnatal care. Mothers’ report was less accurate for intervention coverage provided by health workers around the time of birth. At the same time, events that occurred during postnatal care tended to be reported more accurately.^30 31 34–36^

Studies in India, Kenya and Tanzania that assessed concordance between observer and women’s reports about negative maternity care events found that observers reported labour and childbirth negative maternity care events more frequently than women. The authors attributed the discordance to a range of issues including women underreporting negative maternity care experiences because of social norms, power dynamics, recall issues and mother’s expectation of care.^37–40^

Our findings may also be affected by underreporting of service quality failure (ie, negative maternity care experience) in the exit survey. In response to the discordance observed between observers and women’s self-report of disrespect and abuse Freedman *et al*,^37^ contemplates whose view is ‘correct’ or true—women’s self-report or observer report? Whether the divergence comes from a measurement error or bias? Our findings add to this discussion calling into question the appropriateness of the concept of validity against a reference standard measure for experience of care measures.

We observed an association between mother’s educational status and their reporting consistency, with being educated having a negative effect on agreement between reference standard and repeat interview; educated women became more negative about two positive maternity care experience measures over time. These findings require cautious interpretation due to the wide 95% CIs, for instance, a negative effect on agreement of between 0% and 25% on whether women had a support person present during labour and childbirth, and a negative effect on agreement of between 5% and 30% on whether women were asked which position they would like to deliver in. The literature is conflicting on the recall consistency of women with more education relative to non-educated women. More educated women are more likely to consistently recall measures such as birth weight or gestational age. But, they are also more likely to be more informed and have higher expectations of care quality, greater empowerment to report abuse or underestimate their positive care experiences.^41–45^

### Strengths and limitations

This study makes an important contribution to the literature evaluating measurement methods for tracking positive and negative maternity care experience. It suggests that it is possible to reach a diverse sample of mothers using telephone survey methods, achieving comparable characteristics to an exit survey sample in this setting. Regarding the reference standard, we used exit survey responses from women as they left facilities after childbirth, a ‘best case’ scenario in terms of recall consistency considering mothers were interviewed shortly following facility childbirth.^39^ However, any reference standard is subject to some degree of measurement error, depending on the context and the method used to obtained it.^31^ Women are likely to underestimate the prevalence of negative maternity care experience events and overestimate the prevalence of positive maternity care experience events when self-reporting, possibly due to the normalisation of certain negative maternity care practices or the fear of retaliation from the providers.^37 38 41 46^ Regarding the follow-up telephone interview measure, we cannot know what influence a 14 month recall period had on accuracy. Studies using similar criterion validity methods with 13–15 months recall have previously been conducted.^35^ And 14-month recall is less than that of established household survey programmes which ask women to report on childbirth events that occurred in the preceding 2 or 5 years. To our knowledge, published validation studies to date have reported no relationship between recall period and accuracy of RMNCH indicators.^47–49^ Certainly, questions on experience of care are sensitive and prone to social desirability bias and to try to minimise this effect we used only women as data collectors who received intensive training, and improved clarity of questions through extensive piloting. Regarding our study sample, the study was conducted in 10 PHC facilities and nearly half of the mothers had no formal education, our findings may be more reflective of this type of population, and less generalisable to mothers with higher levels of education, or mothers who deliver in other types of facilities or at home. Our size of sample and the relatively low prevalence for some indicators meant that we were not able to disaggregate findings by the sociodemographic characteristics of respondents.

## Conclusions

Our telephone interview method yielded similar sample characteristics as exit interviews, suggesting that telephone interviews are an interesting option to consider in this study setting. However, the follow-up telephone interviews did not yield the same results about experience of childbirth care as the exit interviews at the time of discharge. It may be that telephone interviews do not generate valid measures for tracking and improving facility-based childbirth experiences. But alternative hypotheses should be considered including that women’s reported experience may legitimately change over time and women’s confidence to report their experiences may differ by place of interview.

## Data Availability

Data are available upon reasonable request.
