# Supplementary material for: To call or not to call: exploring the validity of telephone interviews to derive maternal self-reports of experiences with facility childbirth care in northern Nigeria
Source: BMJ Glob Health. 2022 Mar 16;7(3):e008017. doi: 10.1136/bmjgh-2021-008017 (PMC8928249; doi:10.1136/bmjgh-2021-008017)
Supplement: Supplementary data [file bmjgh-2021-008017supp001.pdf]

Table S1. Positive and negative maternity care experience questions

**Positive maternity care experience questions**
I would like to ask you some questions about your experience during your most recent labour and childbirth.
Were you respectfully greeted by health workers when they first saw you?
Were you encouraged to have a support person present during labour and childbirth?
Were you encouraged to have some light food during labour and delivery?
Were you encouraged to move and change position during labour?
Did you have a support person present during labour and childbirth?
Were the steps involved in every examination during labour and delivery explained to you?
Were you encouraged to ask any questions?
Were you asked which position you would like to deliver in?
**Negative maternity care experience questions**
During your most recent childbirth, did you experience any of the following?
Being beaten, pushed, pinched, slapped, or poked during labour and childbirth?
Being physically restrained, tied, or gagged during labour and childbirth?
Sexually abuse, touched inappropriately or rape during labour and childbirth?
Birth attendant used harsh or rude language, judgmental, or accusatory comments during labour and childbirth?
Birth attendant used threats of withholding treatment or blamed you for poor birth outcomes during labour and childbirth?
Being discriminated against based on ethnicity/race/religion/income/HIV/age during labour and childbirth?
Painful vaginal exams (not acknowledging your discomfort, pain, or refusal to provide pain relief) during labour and childbirth?
Skilled attendant absent at time of childbirth?
Birth attendant performed unconsented surgical operations?
Birth attendant dismissed your concerns during labour and childbirth?
Language and interpretation issues during labour and childbirth?
Poor staff attitudes during labour and childbirth?
Lack of supportive care from health workers during labour and childbirth?
Denied birth companion or lack of birth companions during labour and childbirth?
Denied food, fluids, or mobility during labour and childbirth?
Birth attendant did not respect your choice of preferred birth positions?
Detained in a facility for failure to pay for services?
Lack of privacy during labour and childbirth?
